# Phylogeography of *Scarturus williamsi* and Climate Change Impacts: Genetic Diversity and Projected Habitat Loss in Anatolia

**DOI:** 10.3390/biology14091184

**Published:** 2025-09-03

**Authors:** Zeycan Helvacı, Ercüment Çolak

**Affiliations:** 1 Department of Biology, Faculty of Science and Letter, Aksaray University, 68100 Aksaray, Turkey; 2 Department of Biology, Faculty of Science, Ankara University, 06100 Ankara, Turkey

**Keywords:** jerboa, species distribution modeling, mitochondrial DNA, climate vulnerability, Türkiye

## Abstract

*Scarturus williamsi* (Williams’ jerboa) is a medium-sized, semi-fossorial rodent endemic to steppe ecosystems across Anatolia, Iran, and Azerbaijan, with specialized habitat requirements in semi-arid continental environments. This study integrates a mitochondrial DNA analysis with species distribution modeling to assess the species’ evolutionary structure and vulnerability to future climate change. The phylogeographic analysis and species distribution modeling reveal the evolutionary history and climate vulnerability of *Scarturus williamsi* across Anatolia and adjacent regions. The mitochondrial DNA analysis of 98 individuals demonstrates exceptional haplotype diversity (Hd = 0.9896), with 90 unique haplotypes and complete regional isolation, indicating pronounced population structuring across five evolutionary lineages: Central Anatolia, Eastern Anatolia, Aegean, Black Sea, and Azerbaijan–Iran. The Iran–Azerbaijan lineage exhibits the deepest evolutionary divergence, while Eastern Anatolia functions as the primary Anatolian refugium and Central Anatolia as the secondary refugial center. The strong isolation by distance (r = 0.735, *p* < 0.001) across ~2500 km explains 54.0% of the genetic variation, with the hierarchical structure reflecting greater Iran–Turkey isolation than intra-Turkish differentiation. The species distribution modeling identifies the Mean Temperature of Driest Quarter (bio9) and the Mean Diurnal Range (bio2) as primary habitat determinants, with bimodal preferences reflecting highland versus steppe adaptations. Climate projections reveal severe vulnerability with habitat losses of 63.69–98.41% by 2081–2100 across emission scenarios. SSP3-7.0 represents the most catastrophic scenario, with a severe habitat reduction (98.41% loss), while even optimistic scenarios (SSP1-2.6) project a 60–70% habitat loss. All scenarios show accelerating degradation through mid-century, with the steepest losses occurring between 2041 and 2080. Projected eastward shifts face constraints from the Anatolian Diagonal, limiting the climate tracking capacity. Despite occupying open landscapes, *S. williamsi* exhibits exceptional sensitivity to climate change, with Anatolian refugial areas representing critical diversity centers facing substantial degradation. Results provide baseline genetic structure and climate vulnerability information for understanding climate impacts on *S. williamsi* and Irano–Anatolian steppe fauna.

## Simple Summary

This study integrated genetic analysis and species distribution modeling to assess the climate vulnerability of *Scarturus williamsi*, a rodent endemic to Türkiye and adjacent regions. Results indicate that substantial portions of the species’ current habitat may become unsuitable under future scenarios.

## 1. Introduction

The Anatolian Peninsula (approximately 756,000 km^2^) occupies a unique biogeographic position at the intersection of three biodiversity hotspots: the Mediterranean, Caucasus, and Irano–Anatolian regions. This convergence has created extraordinary taxonomic diversity and high levels of endemism, with over 15 endemic mammal species documented in the region [1,2,3]. The region’s complex topography has played a crucial role in shaping biodiversity patterns and facilitating speciation processes [1,4]. A particularly important geographic feature is the Anatolian Diagonal, a major biogeographic boundary that runs northeast to southwest across Turkey, separating the more humid northern and western regions from the continental interior steppes and creating a steep environmental gradient in Temperature Seasonality [1,5,6]. The Anatolian Diagonal has consistently influenced phylogeographic patterns across diverse taxonomic groups, including mountain frogs [7], banded newts [8], rodents [9], and the grasshoppers [10], acting both as a dispersal barrier for many species and as a transitional zone between different bioclimatic regions. The region’s mountains have functioned as refugia during climatic fluctuations, allowing for the accumulation of intraspecific genetic diversity and the formation of local endemic species [4,11]. However, the same geographic complexity that promoted diversification may also constrain species’ ability to track suitable climates under current climate change scenarios. Specifically, the mountain ranges and topographic barriers that historically isolated populations and facilitated speciation now act as physical obstacles preventing the rapid dispersal and migration needed to follow shifting climate zones. This creates a paradox where the geographic features that enhanced evolutionary diversification may limit adaptive responses to rapid contemporary climate change.

*Scarturus williamsi* Thomas, 1897 (Williams’ jerboa) is a medium-sized jerboa endemic to Anatolian steppe ecosystems and adjacent regions. Previously classified within *Allactaga* [12,13], this species was historically thought to include an isolated population in Afghanistan, but molecular systematic investigations have since determined that population represents a distinct species, *Scarturus caprimulga* [14]. As a herbivorous rodent feeding mainly on underground plant parts and occasionally on insects in semi-arid steppe habitats, *S. williamsi* likely contributes to ecosystem functionality through seed dispersal mechanisms common to desert-adapted rodents [15], while serving as prey for regional steppe predators. The species represents an ideal model system for investigating phylogeographic patterns and climate vulnerability in arid-adapted small mammals. A recent phylogeographic analysis has revealed complex intraspecific structures encompassing five distinct evolutionary lineages: W1 (*S. w. schmidti* and *S. w. williamsi* from Eastern Anatolia, Iran, and Azerbaijan), W2 (*S. w. laticeps* from Eastern Anatolia), W3 (Western Anatolian populations), W4 (Central Anatolian specimens), and W5 (unique Niğde lineage) [16]. This substantial population subdivision appears to be shaped by geographic and ecological constraints. Recent phylogenetic work within Allactaginae has clarified intergeneric relationships and species delimitation within *Scarturus*, while reconstructing historical range dynamics and temporal diversification patterns [14]. Although recent studies (such as [17]) have improved our knowledge of jerboa relationships in general, we still lack detailed information about how *S. williamsi* evolved and how it responds to environmental changes, including its habitat needs and vulnerability to climate change.

This complex phylogeographic structure and pattern of genetic divergence provides an important framework for understanding how *S. williamsi* populations might respond to environmental pressures. As we consider the implications of this genetic structure, it is important to contextualize it within the broader challenges facing biodiversity globally. Climate change represents one of the most pressing challenges to global biodiversity, with species facing unprecedented rates of environmental transformation that threaten their persistence across ecosystems worldwide [18]. Small mammals, particularly those with specialized habitat requirements and limited dispersal capabilities, are among one of the most vulnerable taxonomic groups to these rapid environmental changes [19,20].

Although *S. williamsi* is currently classified as ‘Least Concern’ by the IUCN Red List (global assessment), the Mediterranean Regional Assessment lists the species as ‘Near Threatened’, reflecting regional population pressures. This discrepancy highlights that the global assessment may not adequately capture the species’ vulnerability to rapid climate change. Contrary to expectations that open landscape specialists might benefit from global warming, desert-adapted small mammals face a paradoxical challenge: they already inhabit environments near their physiological tolerance limits for temperature and aridity [21]. Jerboa species show relatively narrow thermoregulatory windows and potentially limited behavioral plasticity under extreme heat stress [19]. Arid ecosystems are projected to experience some of the most severe impacts from rising temperatures and altered precipitation regimes, with potential increases in temperature extremes and shifts in resource availability that might exceed species’ adaptive capacity [22,23,24]. Desert-adapted species like *Welwitschia mirabilis* Hook.f. Welwitch, F.M.J. (1859) in the Namib Desert already show population declines that appear consistent with climate change projections, potentially serving as bioindicators for broader ecosystem effects [25]. Recent studies of desert mammal communities in the Mojave Desert show that while some species have maintained stability through microhabitat buffering strategies like burrowing, the magnitude of projected future warming may exceed these adaptive capacities, particularly for species with specialized habitat requirements [26]. Such differential responses among desert mammals potentially serve as indicators for broader ecosystem effects and highlight the importance of species-specific vulnerability assessments.

The integration of species distribution modeling (SDM) and molecular phylogeographic approaches has emerged as a powerful tool for understanding species’ responses to past and future climate changes [27,28,29]. This combined approach allows us to identify refugia, assess population connectivity, and evaluate species vulnerability to climate change with greater accuracy and insight. SDM combined with phylogeographic data can reveal historical refugia and validate assumptions of niche conservatism [30]. For example, studies on mammals have successfully applied these concepts, such as the research on wild boars (*Sus scrofa* Linnaeus, 1758) that used distribution modeling to demonstrate niche conservatism across invasion ranges, validating predictions based on ancestral climate preferences [31]. Similarly, research on *Triturus* (Rafinesque, 1815) newts used SDM and mitochondrial phylogeography to reconstruct glacial refugia and post-glacial expansion patterns, providing a more complete biogeographical scenario than either method alone [32]. The integration of these complementary approaches allows us to identify refugial areas, assess population connectivity, and evaluate species vulnerability to climate change while accounting for both contemporary ecological constraints and historical evolutionary processes.

*Scarturus williamsi* populations exhibit strong phylogeographic structuring aligned with historical refugial regions, and their suitable habitats may undergo substantial contraction under future climate scenarios. This study aims to integrate species distribution modeling with mitochondrial DNA analysis to evaluate the phylogeographic structure and climate vulnerability of *S. williamsi*. Building on existing genetic frameworks, the objectives are as follows: (1) characterize the current geographic distribution and genetic structure of *S. williamsi* across its range using comprehensive phylogenetic analyses including uncertainty assessments; (2) identify key environmental variables controlling habitat suitability; (3) model potential range shifts under multiple future climate scenarios; and (4) evaluate the species’ vulnerability to climate change and identify climatically stable areas that may serve as refugia. By combining species distribution modeling with genetic data, this research provides critical baseline information for evaluating potential impacts of climate change on *S. williamsi* and related steppe-adapted taxa in Türkiye and adjacent regions.

## 2. Materials and Methods

All statistical analyses, geospatial processing, and data visualizations were conducted using R v4.3.0 [33] in a fully reproducible workflow.

### 2.1. Species Occurrence Data and Study Area

**Figure 1:**
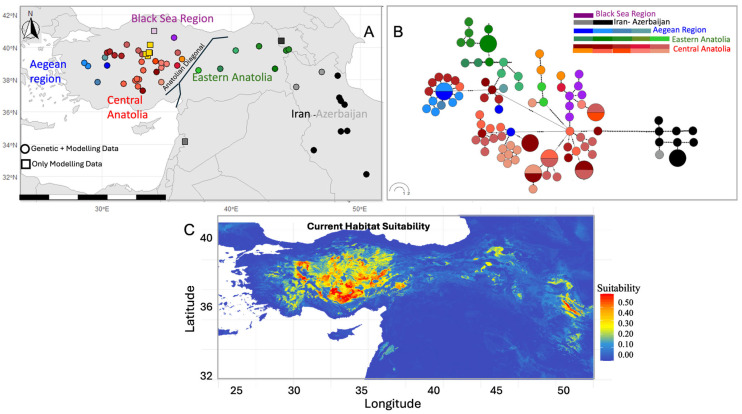
(**A**) Geographic distribution of *Scarturus williamsi* across Turkey and neighboring countries based on species distribution modeling (SDM) and genetic sampling. Circles indicate localities with both SDM presence and genetic material; squares indicate localities with only SDM presence. Localities are colored by region: red hues for Central Anatolia, green for Eastern Anatolia, blue for the Aegean region, purple for the Black Sea region, and black/gray for neighboring countries. (**B**) Haplotype network of *Scarturus williamsi* populations, colored by geographic region. Node sizes are proportional to haplotype frequency; black dots represent mutational steps. (**C**) Current habitat suitability map for *Scarturus williamsi* based on Random Forest modeling using WorldClim bioclimatic variables. Warmer colors (red to yellow) indicate higher habitat suitability; cooler colors (blue) represent low or unsuitable areas.

Occurrence records of *S. williamsi* were compiled through a comprehensive review of literature and biodiversity databases, including GBIF.org [34] and IUCN [35]. To ensure completeness, all known synonyms (e.g., *Allactaga williamsi*, ‘Arap tavşanı’, ‘Williams Jerboa’) were included in the search. Georeferenced records were validated and manually corrected or excluded if coordinates were deemed unreliable. The study region spans 25–73° E longitude and 27–42° N latitude, encompassing all confirmed records (Figure 1A).

A basemap was generated using the rnaturalearth package v1.0.1 [36]. Species distribution was visualized by plotting occurrence points on this basemap with ggplot2 v3.5.2 [37].

### 2.2. Genetic Analysis

Mitochondrial cytochrome *b* sequences (888 bp) from 98 *S. williamsi* individuals were analyzed to assess genetic diversity and population relationships across biogeographic regions. Sequences were obtained from GenBank nucleotide database (accession numbers documented in Table S1), representing comprehensive sampling across five distinct biogeographic populations: Central Anatolia (n = 55), Eastern Anatolia (n = 16), Aegean region (n = 11), Iran–Azerbaijan (n = 10), and Black Sea region (n = 6). Unique haplotype determination employed the haplotype() function within the pegas package v1.1 [38], implementing nucleotide sequence comparison algorithms to identify distinct mitochondrial lineages and calculate haplotype frequencies across sampling localities. Comprehensive population genetic diversity metrics were computed for each biogeographic region using the pegas and ape packages, including (1) number of unique haplotypes (Nh); (2) haplotype diversity (Hd), quantifying the probability that two randomly chosen haplotypes differ [39]; (3) nucleotide diversity (π), measuring average number of nucleotide differences per site between sequences; (4) number of segregating sites (S), representing polymorphic positions; (5) Watterson’s theta (θw), estimating the population mutation parameter [40]; and (6) Tajima’s D statistic for testing neutrality and detecting demographic expansion signatures [41]. Statistical significance of Tajima’s D was assessed using normal approximation with α = 0.05.

Phylogenetic relationships among haplotypes were visualized using the haploNet() function in pegas package, constructing statistical parsimony networks to display evolutionary connections between mitochondrial lineages.

Phylogenetic relationships and topological uncertainty were systematically evaluated through Bayesian inference methodologies implemented within the R computational framework. Sequence alignment integrity was initially assessed through visual inspection using the image.DNAbin() function to verify nucleotide data quality and detect potential alignment artifacts prior to phylogenetic reconstruction.

Evolutionary relationships were reconstructed using BEAST2 v2.6.0 phylogenetic software, accessed through the babette package interface v2.3.2 [42] in R. Bayesian analysis employing the bbt_run_from_model() function with default model parameters, utilizing the aligned FASTA sequence dataset as input for Markov Chain Monte Carlo (MCMC) posterior sampling of phylogenetic trees and associated parameters.

Topological uncertainty across the posterior distribution of phylogenetic trees was visualized using DensiTree methodology [43] implemented through the plot_densitree() function. Tree cloud visualization incorporated 1001 posterior trees sampled from generations 9000–10,000 of the MCMC chain, with transparency scaling (alpha = 0.03) to represent posterior probability density, fixed branch scaling, and standardized graphical parameters for consistent visual representation of phylogenetic uncertainty patterns. Geographic origin of terminal taxa was color-coded to facilitate identification of biogeographic clustering patterns within the evolutionary framework.

To test for isolation by distance (IBD), pairwise genetic distances between all sampling localities were calculated using the Kimura 2-parameter (K2P) substitution model implemented in the R package ‘ape’ v5.0 [44]. The K2P model accounts for different rates of transitions and transversions and is widely used for intraspecific phylogenetic analyses [45]. For localities with multiple individuals, mean genetic distances were computed from all pairwise comparisons between localities to obtain representative inter-population genetic distances.

Geographic distances between sampling sites were calculated as great circle distances using the ‘geosphere’ package v1.5-20 in [46], which accounts for the curvature of the Earth’s surface. The significance of the correlation between genetic and geographic distance matrices was tested using a Mantel test with 999 permutations implemented in the ‘vegan’ package v2.6-4 [47]. The Mantel test is appropriate for this analysis as it accounts for the non-independence of distance matrices, preventing the use of standard parametric correlation methods [48,49].

The strength of the IBD pattern was assessed using Pearson’s correlation coefficient, and statistical significance was determined at α = 0.05. The coefficient of determination (r^2^) was calculated to estimate the proportion of genetic variation explained by geographic distance.

### 2.3. Species Distribution Modeling, Future Projections, and Climate Impact Assessment

Climatic predictor variables were obtained from the WorldClim v2.1 [50] database at a 30 arc-second spatial resolution (~1 km^2^ at equator). Future climate projections utilized statistically downscaled CMIP6 global circulation model outputs from MIROC6 Earth System Model across four Shared Socioeconomic Pathways (SSP1-2.6, SSP2-4.5, SSP3-7.0, SSP5-8.5) spanning four temporal projection intervals (2021–2040, 2041–2060, 2061–2080, and 2081–2100), which represent standard near-term, mid-century, late-century, and end-of-century periods commonly used in IPCC (Intergovernmental Panel on Climate Change) assessments to capture progressive climate trends.

From the initial set of 19 bioclimatic variables obtained from WorldClim, a rigorous selection process was conducted. Ecological relevance for *S. williamsi* was first assessed, followed by statistical screening for multicollinearity using the vifcor function (usdm package v2.1-7 [51]), with correlation threshold of 0.8. Random Forest (RF) algorithm within the sdm package v1.2-55 [52] employed subsampling data partitioning with 70% training and 30% testing allocation across 10 replicate model iterations to quantify prediction uncertainty. Pseudo-absence generation utilized spatially random background point sampling (method = ‘gRandom’, n = 1000) distributed across the study extent. Model ensemble predictions implemented weighted averaging based on True Skill Statistic performance metrics (method = ‘weighted’, stat = ‘tss’, opt = 2) via the ensemble() function, generating consensus habitat suitability surfaces with integrated uncertainty quantification.

Predictive accuracy assessment incorporated multiple complementary metrics: Area Under the Receiver Operating Characteristic Curve (AUC) for discrimination capacity, Pearson correlation coefficient (COR) for continuous prediction accuracy, True Skill Statistic (TSS) adjusting for prevalence effects, and explained deviance for goodness-of-fit evaluation. All these approaches ensured the selection of biologically meaningful predictors while maintaining statistical robustness for modeling current and future distributions of the target species under climate change scenarios. The analysis covered the entire global range of the species, with the exception of Afghanistan, which was excluded due to its geographic isolation and lack of connectivity with other suitable habitats. According to Lebedev, Shenbrot [14], the jerboa population in Afghanistan is not *S. williamsi* but rather a distinct species, *S. caprimulga*. Distribution maps were produced for each scenario, and changes between the baseline and 2100 were analyzed to assess potential range shifts under climate change.

Habitat loss was quantified by comparing current suitable habitat with future projections. A suitability threshold of 0.3 was applied to convert continuous habitat suitability maps to binary presence/absence maps, representing areas with ≥30% habitat suitability. This threshold captures moderate to high-quality habitats while including potential dispersal corridors and marginal habitats that may be important for species persistence under climate change.

## 3. Results

### 3.1. Genetic Diversity and Phylogeographic Structure

The analysis of cytb of *S. williamsi* revealed an exceptionally high genetic diversity across all sampled biogeographic regions (Table 1 and alignment of sequences, with nucleotide positions given in Figure S1). The complete dataset yielded 90 unique haplotypes with an overall haplotype diversity of 0.9896, indicating that nearly every individual possessed a distinct mitochondrial lineage. No haplotypes were shared between biogeographic regions, indicating a strong population structure characterized by complete genetic isolation between regions.

All populations exhibited negative Tajima’s D values (range: −0.231 to −1.500), though none deviated significantly from neutrality (all *p* > 0.05). High θw values in Central (22.730) and Eastern Anatolia (22.301) combined with high Hd and a non-significant Tajima’s D strongly support these regions as primary refugia that maintained large, diverse populations throughout glacial–interglacial cycles. Eastern Anatolia showed values closest to neutrality (D = −0.231, *p* = 0.817), potentially indicating a more stable demographic history. The peripheral populations (Aegean and Black Sea regions) exhibited lower mutational diversity (θw = 9.901 and 7.007, respectively) and more negative Tajima’s D values, consistent with the secondary colonization from the primary refugial areas (Table 1).

The haplotype network analysis revealed that Central Anatolian populations formed the largest cluster, with multiple high-frequency haplotypes occupying central network positions (Figure 1B), indicating ancestral diversity. Eastern Anatolian haplotypes formed a distinct cluster connected to the main network through single mutational steps, suggesting recent divergence. Aegean region haplotypes clustered separately as a peripheral group, while Iran–Azerbaijan populations appeared as a completely isolated cluster separated by multiple mutational steps, indicating long-term geographic isolation. Despite evolutionary connectivity through short mutational pathways, no haplotypes were shared between regions, confirming complete contemporary isolation.

**Figure 2:**
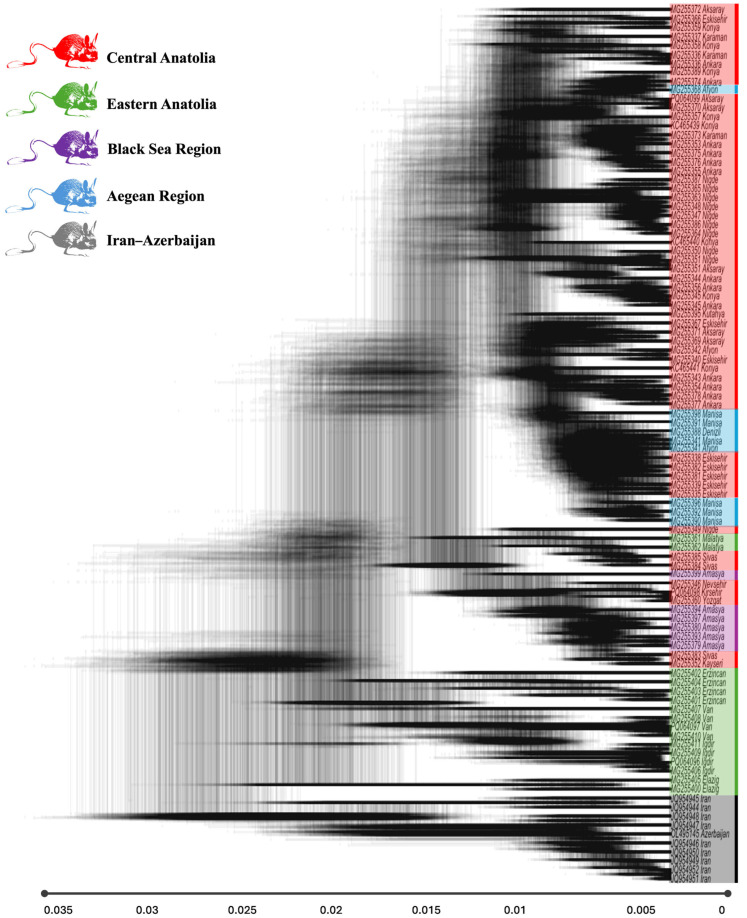
DensiTree representation of *Scarturus williamsi* individuals based on phylogenetic analysis. Each line represents a sampled gene tree, with darker areas indicating higher congruence among trees. Terminal labels (tips) are colored according to their geographic origin: red for Central Anatolia, green for Eastern Anatolia, blue for the Aegean region, purple for the Black Sea region, and gray for Iran–Azerbaijan. The plot was configured with high transparency (alpha = 0.03) to effectively display areas of topological congruence and discordance among trees.

The DensiTree analysis revealed substantial phylogenetic uncertainty across *S. williamsi* lineages, with cloud-like tree distributions indicating conflicting phylogenetic signals (Figure 2). Despite this uncertainty, geographic clustering patterns were consistently maintained, supporting strong biogeographic structures. Eastern Anatolian lineages frequently occupied basal positions, consistent with their role as ancient refugial populations. Central Anatolian populations showed variable positions throughout the tree space, reflecting their high mutational diversity and complex refugial history. Iran–Azerbaijan, representing the easternmost populations of *S. williamsi,* consistently occupied the most basal positions with the longest branch lengths, indicating the deepest evolutionary divergence and long-term isolation from Turkish populations.

The combination of the network centrality in Central Anatolia and the basal phylogenetic positioning in Eastern Anatolia revealed contrasting evolutionary patterns: Eastern Anatolia exhibited the highest nucleotide diversity (π = 0.023761), with a basal phylogenetic placement, while Central Anatolia showed network centrality with moderate negative Tajima’s D values that remained non-significant (D = −1.475, *p* = 0.140), consistent with refugial stability. This pattern extended across the geographic diversity gradient, with nucleotide diversity decreasing from east to west: Eastern Anatolia (π = 0.023761) > Iran–Azerbaijan (π = 0.016692) > Central Anatolia (π = 0.014861) > Aegean region (π = 0.007535). The geographic distribution of the genetic diversity showed clear patterns: populations from the eastern highlands maintained the highest nucleotide diversity values and occupied basal phylogenetic positions, while western populations showed a reduced diversity despite network centrality, supporting a model of ancient refugial populations in eastern regions with secondary colonization westward.

**Figure 3:**
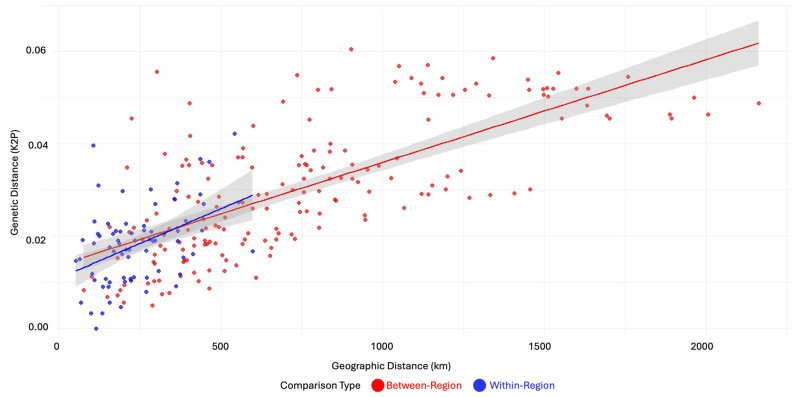
Regional isolation by distance analysis for *S. williamsi* across Anatolia and adjacent regions. Scatterplot shows pairwise comparisons between 23 sampling localities (72 within-region comparisons in blue, 181 between-region comparisons in red) with genetic distance (Kimura 2-parameter model) plotted against geographic distance. Solid lines represent linear regression trends for each comparison type.

A significant overall isolation by distance (IBD) pattern was detected (Mantel test: r = 0.735, *p* < 0.001), with the geographic distance explaining 54.0% of the genetic variation among populations. The hierarchical analysis revealed distinct processes operating at different spatial scales. Both comparison types showed significant positive correlations (within-region: r = 0.44, *p* < 0.001; between-region: r = 0.70, *p* < 2.6 × 10^−28^), though between-region comparisons exhibited an approximately 1.6× stronger isolation by distance (Figure 3). Between-region comparisons showed steeper genetic differentiation with distance than within-region comparisons, suggesting the presence of historical or topographic barriers to gene flow, consistent with the deep phylogeographic structuring observed in the haplotype data.

### 3.2. Species Distribution Modeling

The Random Forest model demonstrated a robust performance across all evaluation metrics. The model validation indicated a satisfactory predictive accuracy with an AUC value of 0.84, reflecting a strong discrimination between suitable and unsuitable habitats. The TSS value of 0.64 demonstrated a moderate agreement between predicted and observed distributions, while the correlation coefficient of 0.33 indicated a modest but positive correspondence between model predictions and the occurrence data. The model deviance of 0.34 suggested a reasonable model fit, collectively confirming the effectiveness of the Random Forest algorithm for modeling the *S. williamsi* distribution.

**Figure 4:**
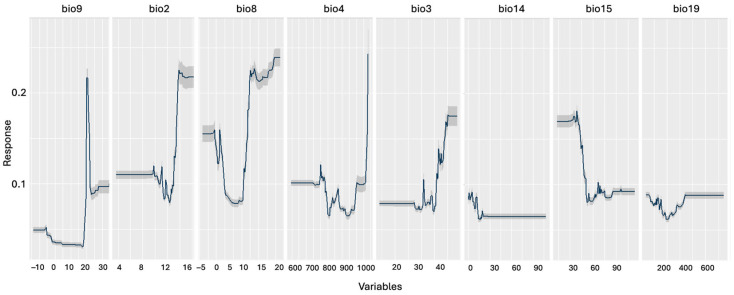
Predicted habitat suitability response curves for *Scarturus williamsi* based on Random Forest species distribution modeling. Response curves show the relationship between environmental variables and predicted habitat suitability while holding all other variables at their mean values. Variables are ordered by decreasing importance scores from the Random Forest model (shown in parentheses): bio9 = Mean Temperature of Driest Quarter (°C); bio2 = Mean Diurnal Range (°C); bio8 = Mean Temperature of Wettest Quarter (°C); bio4 = Temperature Seasonality (standard deviation × 100); bio3 = Isothermality (%); bio14 = Precipitation of Driest Month (mm); bio15 = Precipitation Seasonality (coefficient of variation); and bio19 = Precipitation of Coldest Quarter (mm). Bioclimatic variables represent annual trends, seasonality, and extreme environmental factors.

The permutation-based variable importance analysis revealed distinct contributions of bioclimatic predictors to habitat suitability (Figure 4, Table 2, Figure S2).

Temperature-related variables emerged as primary determinants, with bio9 (Mean Temperature of Driest Quarter) showing the strongest influence (23.5% correlation importance, 8.6% AUC importance). Bio2 (Mean Diurnal Range) ranked second in importance (16.2% correlation importance), followed by Precipitation Seasonality variables bio14 (Precipitation of Driest Month, 10.0%) and bio15 (Precipitation Seasonality, 9.9%). The remaining variables showed progressively lower contributions, with bio19 (Precipitation of Coldest Quarter) exhibiting a minimal influence on model predictions.

Random Forest-derived response curves show the habitat suitability for *Scarturus williamsi* across key bioclimatic variables. Bio9 (Mean Temperature of Driest Quarter, °C) emerged as the most influential predictor, exhibiting a sharp peak at intermediate temperatures (~10–15 °C), which suggests critical thermal constraints during drought periods. Secondary predictors included the following: bio2 (Mean Diurnal Range), showing an optimal suitability at moderate daily temperature fluctuations; bio8 (Mean Temperature of Wettest Quarter), indicating warmer wet-season preferences; and bio4 (Temperature Seasonality), reflecting the adaptation to moderate seasonal variation. Precipitation variables (bio14, bio15, bio19) demonstrated narrower response breadths than thermal variables, with the species preferring intermediate dry-month precipitation (bio14) and low–moderate Precipitation Seasonality (bio15). These patterns collectively suggest that *S. williamsi* is adapted to Central Anatolia’s continental climate, with specific physiological requirements during arid periods that may reflect Pleistocene refugial conditions.

### 3.3. Current Habitat Suitability and Species Distribution

Current optimal habitats (suitability > 0.7) are concentrated in Central and Eastern Anatolia (Figure 1C). Marginal suitability zones (0.4–0.7) occurred in transitional areas within the Aegean region and northern peripheral zones (Amasya and Yozgat).

**Figure 5:**
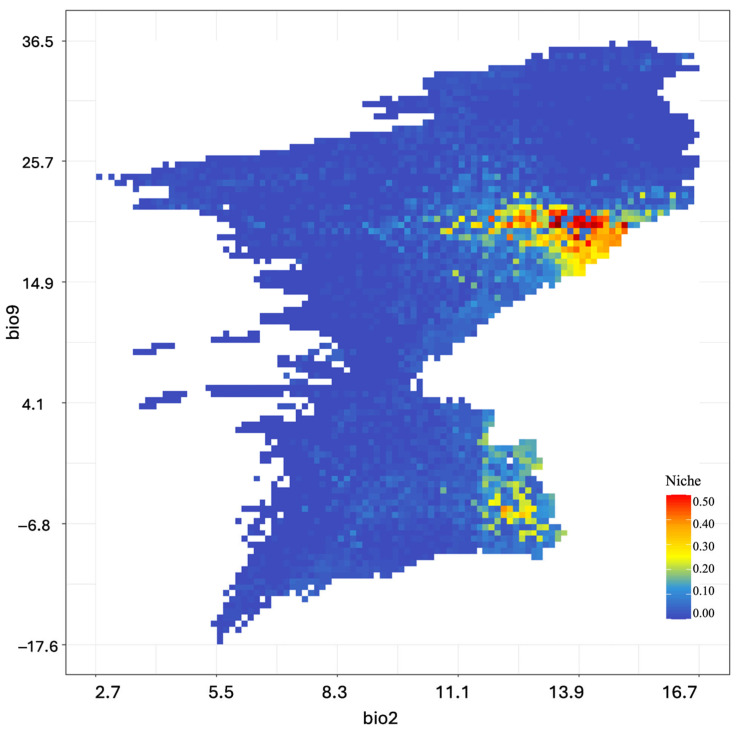
Predicted ecological niche suitability for *Scarturus williamsi* as a function of bio2 (Mean Diurnal Range) and bio9 (Mean Temperature of Driest Quarter).

The analysis of environmental variable responses revealed distinct climate preferences with evidence of population differentiation (Figure 5). For bio9 (Mean Temperature of Driest Quarter), optimal habitat suitability (0.50) occurred between 15 and 25 °C, representing the species’ preference for moderate temperatures during dry periods. A secondary suitability peak (0.30) appeared under colder conditions (−6 to 1 °C), suggesting potential population structures or separate lineages adapted to distinct thermal environments. For bio2 (Mean Diurnal Range), the highest suitability (0.40) was observed between 11 and 14 °C, indicating a preference for moderate daily temperature variation, while a narrower range (12–13.5 °C) showed lower suitability (0.20), reflecting a reduced tolerance for greater diurnal fluctuations.

### 3.4. Future Habitat Projections Under Climate Scenarios

**Figure 6:**
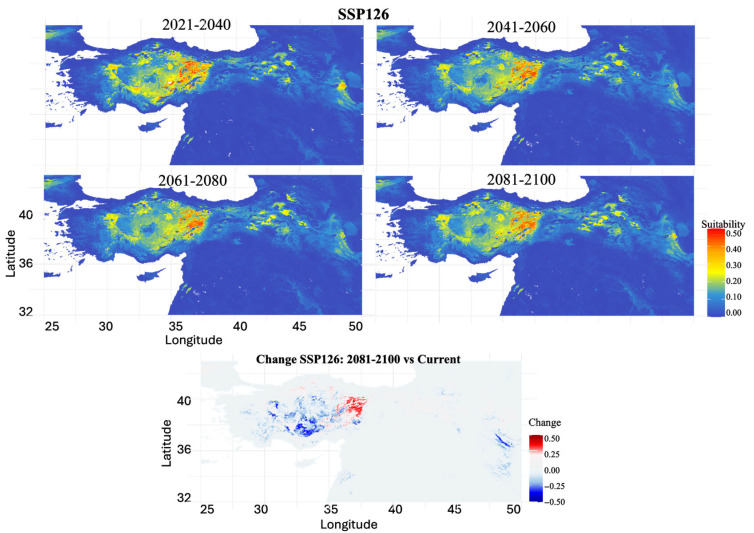
Projected future habitat suitability for *S. williamsi* under the SSP1-2.6 (low emissions) climate scenario. The four upper panels show modeled habitat suitability across four time periods (2021–2040, 2041–2060, 2061–2080, 2081–2100), with warmer colors (yellow to red) indicating areas of high habitat suitability (0.3–0.5) and cooler colors (blue) representing low suitability or unsuitable habitat (0.0–0.2). The bottom panel displays habitat suitability changes between the 2081–2100 projection and current conditions, where red areas indicate suitability gains (+0.5 to +0.25), blue areas show suitability losses (−0.5 to −0.25), and gray areas represent minimal change.

SSP1-2.6 (Low Emissions Scenario): Under low emission projections, the habitat suitability remained relatively stable across the 21st century (Figure 6). The habitat loss increased from 48.24% (2021–2040) to a maximum of 67.81% (2061–2080), followed by a partial recovery to 63.69% by 2081–2100. Central Anatolia consistently maintained core suitable areas, with an evident gradual eastward and northward expansion. The bottom panel revealed suitability gains in Central and cent (Niğde, Kayseri, Sivas) while western regions (Afyon, Manisa) showed reduced suitability.

**Figure 7:**
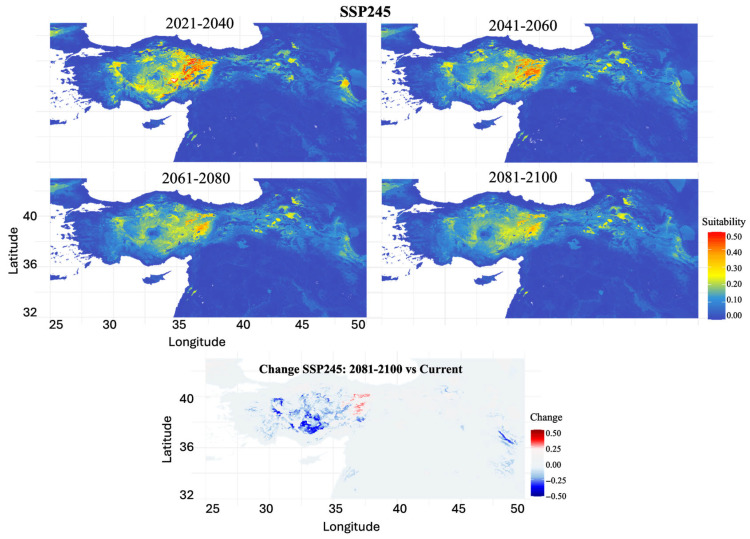
Projected future habitat suitability and potential distributional change for *Scarturus williamsi* under climate scenario SSP245. The four upper panels show modeled habitat suitability across four time periods (2021–2040, 2041–2060, 2061–2080, 2081–2100), with warmer colors (yellow to red) indicating areas of high habitat suitability (0.3–0.5) and cooler colors (blue) representing low suitability or unsuitable habitat (0.0–0.2). The bottom panel displays habitat suitability changes between the 2081–2100 projection and current conditions, where red areas indicate suitability gains (+0.5 to +0.25), blue areas show suitability losses (−0.5 to −0.25), and white areas represent minimal change.

SSP2-4.5 (Moderate Emissions Scenario): Moderate emission projections showed progressive habitat degradation throughout the century (Figure 7). The habitat loss escalated from 46.92% (2021–2040) to 90.27% by 2081–2100, representing severe range contraction. Core suitable areas in Central and Eastern Anatolia persisted through mid-century but showed increasing fragmentation toward 2081–2100. Significant suitability increases were projected for parts of Central and Eastern Anatolia, while coastal regions, particularly western and southern areas, experienced marked declines.

**Figure 8:**
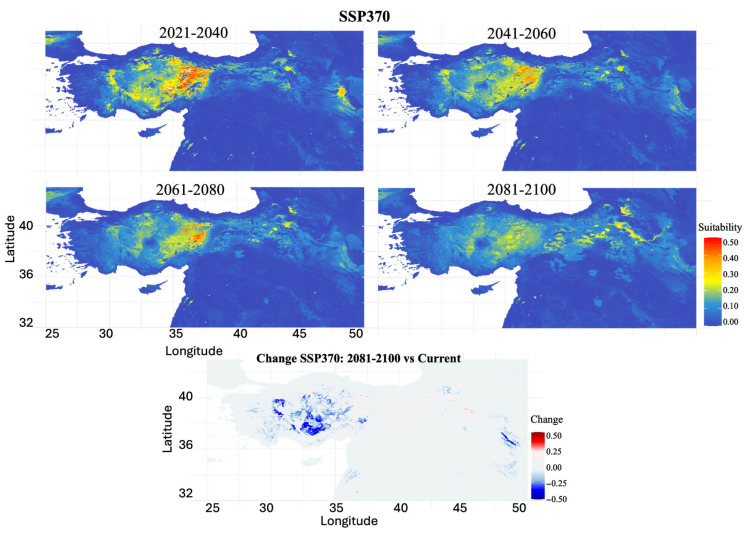
Projected future habitat suitability and potential distributional change for *Scarturus williamsi* under climate scenario SSP370. The four upper panels show modeled habitat suitability across four time periods (2021–2040, 2041–2060, 2061–2080, 2081–2100), with warmer colors (yellow to red) indicating areas of high habitat suitability (0.3–0.5) and cooler colors (blue) representing low suitability or unsuitable habitat (0.0–0.2). The bottom panel displays habitat suitability changes between the 2081–2100 projection and current conditions, where red areas indicate suitability gains (+0.5 to +0.25), blue areas show suitability losses (−0.5 to −0.25), and white areas represent minimal change.

SSP3-7.0 (High Emissions Scenario): High emission scenarios projected the most severe habitat contraction (Figure 8). The habitat loss progressed from 39.76% (2021–2040) to a severe habitat reduction of 98.41% by 2081–2100. Suitable habitats showed clear decline trends throughout the century, with moderately suitable areas in Central Anatolia progressively shrinking and fragmenting. By the final period, only small, isolated patches of low to moderate suitability remained, primarily in Central Anatolia.

**Figure 9:**
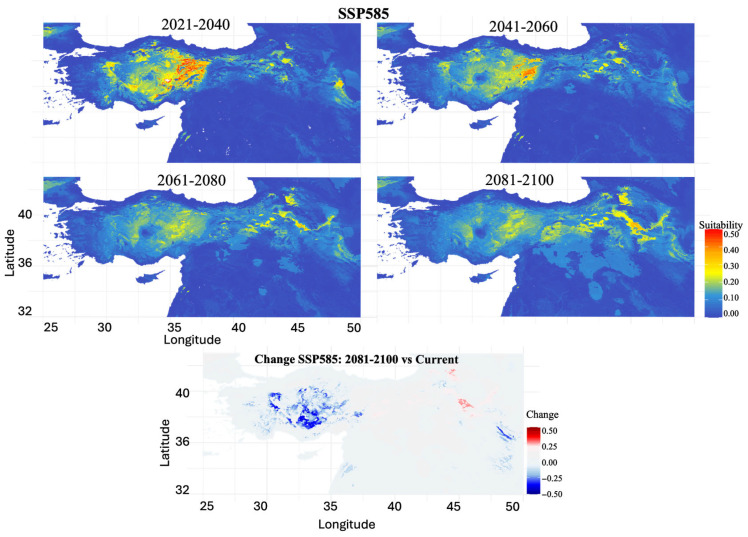
Projected future habitat suitability and potential distributional change for *Scarturus williamsi* under climate scenario SSP585. The four upper panels show modeled habitat suitability across four time periods (2021–2040, 2041–2060, 2061–2080, 2081–2100), with warmer colors (yellow to red) indicating areas of high habitat suitability (0.3–0.5) and cooler colors (blue) representing low suitability or unsuitable habitat (0.0–0.2). The bottom panel displays habitat suitability changes between the 2081–2100 projection and current conditions, where red areas indicate suitability gains (+0.5 to +0.25), blue areas show suitability losses (−0.5 to −0.25), and white areas represent minimal change.

SSP5-8.5 (Extreme Emissions Scenario): Extreme emission projections indicated substantial habitat degradation, with habitat loss increasing from 43.46% (2021–2040) to 97.34% (2061–2080), followed by a slight recovery to 93.14% by 2081–2100 (Figure 9). The primary suitable habitat in Central Anatolia underwent a progressive reduction and fragmentation, with large areas showing significant suitability decreases. Minimal scattered patches indicated slight suitability increases in northeastern Anatolia by the century’s end, though these were vastly outweighed by extensive habitat losses.

Habitat loss projections revealed a clear emissions-dependent gradient (Table 3), with low emission scenarios (SSP1-2.6) maintaining approximately 60–70% habitat loss by the century’s end, while moderate to extreme scenarios (SSP2-4.5, SSP3-7.0, SSP5-8.5) projected catastrophic losses exceeding 90%. SSP3-7.0 emerged as the most severe scenario, projecting a severe habitat reduction (98.41% loss) by 2081–2100. All scenarios showed accelerating habitat loss through the mid-century, with the steepest degradation occurring between 2041 and 2080. Central Anatolian core areas demonstrated a relative persistence across scenarios, while peripheral and transitional zones showed consistent vulnerability to climate-driven habitat loss.

## 4. Discussion

### 4.1. Phylogeographic Structure and Evolutionary History

The phylogeographic analysis of *Scarturus williamsi* reveals a complex pattern of genetic diversity that reflects the intricate geological and climatic history of the Anatolian Peninsula. The exceptionally high haplotype diversity (Hd = 0.9896) combined with the complete absence of shared haplotypes between biogeographic regions demonstrates a remarkable level of population structure for a small mammal species. This pattern suggests long-term isolation between regional populations, likely maintained by persistent biogeographic barriers and a limited dispersal capacity [53,54].

This exceptional level of haplotype uniqueness, with each biogeographic region harboring exclusively private haplotypes, indicates evolutionary differentiation that extends beyond current ecological timescales. However, phylogenetic relationships reveal a hierarchical population structure rather than uniform regional isolation, with varying degrees of evolutionary independence among different regional comparisons [55,56,57]. The non-significant but consistently negative Tajima’s D values across all populations (−0.231 to −1.500) support demographic stability rather than recent expansion, which is consistent with long-term refugial persistence [58,59].

### 4.2. Refugial Dynamics and Phylogenetic Uncertainty

Despite this uncertainty, geographic clustering patterns show variable strength, reflecting complex phylogenetic relationships that result from historical gene flow followed by complete isolation. Nevertheless, the phylogenetic uncertainty suggests that signatures of historical connectivity remain in the genetic data [14,60,61].

The clearest biogeographic pattern involves Iranian and Azerbaijani populations, which consistently occupy the most divergent positions across the posterior tree distribution, indicating substantial genetic differentiation from Turkish populations. This strong separation pattern suggests a deep intraspecific divergence that reflects the complex biogeographic history of the broader Irano–Anatolian region [62]. Studies on brown frogs [63], pit vipers [64], and racerunner lizards [65] consistently show that Iranian and Turkish populations often occupy divergent positions in phylogenetic trees. This divergence likely reflects the geographic isolation imposed by the Armenian Highland and the Zagros Mountains, which have acted as effective barriers to gene flow between western Iranian and Turkish populations throughout evolutionary history.

Within the Turkish populations, Central and Western Anatolian samples show extensive phylogenetic mixing across posterior tree distributions, with samples from these regions frequently clustering together despite their geographic separation. This pattern suggests a shared evolutionary history and a recent divergence within the past ~800,000 years, corresponding to divergence patterns documented within *Scarturus* species groups, where the most closely related taxa separated during the Middle Pleistocene [14]. This temporal framework contrasts with the stronger isolation between Iranian and Turkish populations [66,67]. The hierarchical biogeographic structure reflects different levels of population differentiation across the species’ range, with the Iran–Turkey division representing deeper evolutionary separation than relationships among Turkish regional populations [54,57].

Within Turkish populations, Eastern Anatolia represents the primary refugium based on its highest nucleotide diversity (π = 0.023761), most neutral demographic signatures (Tajima’s D = −0.231, *p* = 0.817), and evidence of maintaining the largest and most stable populations throughout Quaternary climatic oscillations. Central Anatolia served as a secondary refugium, evidenced by its central position in haplotype networks, highest segregating site count (S = 104), and elevated Watterson’s theta values (θw = 22.730), which is consistent with paleoclimatic evidence [68,69]. Eastern Anatolian samples show varying degrees of phylogenetic resolution, with the genetic differentiation observed between Eastern Anatolian populations and other lineages likely reflecting prolonged isolation in highland refugia, where cooler temperatures and distinct precipitation regimes may have favored local adaptation. The complex topography of Eastern Anatolia, characterized by high mountain ranges and deep valleys, has promoted population fragmentation and localized adaptation consistent with Middle Pleistocene divergence timescales observed in closely related jerboa taxa [14].

Central Anatolia’s role as a secondary refugium is evidenced by its central position in haplotype networks, highest segregating site count (S = 104), and elevated Watterson’s theta values (θw = 22.730). Central Anatolian localities display remarkable phylogenetic diversity, with samples from the same localities often appearing in different parts of the tree. The highest genetic diversity observed in the Konya–Aksaray (Central Anatolia) region suggests this area has served as a long-term refugium, which is consistent with paleoclimatic evidence indicating the persistence of steppe habitats in Central Anatolia throughout Quaternary climate oscillations [68], reflecting Early Pleistocene radiation patterns characteristic of the *S. euphraticus* species group [14].

Western Anatolian samples exhibit relatively lower phylogenetic diversity and form more cohesive clusters compared to Central Anatolian populations. This pattern suggests the more recent colonization of western regions, possibly during the post-glacial expansion from eastern refugia. The reduced topographic complexity in Western Anatolia may have facilitated gene flow, leading to the observed genetic homogeneity.

### 4.3. Integration of Genetic and Ecological Niche Data

The identification of Central Anatolia as both a genetic diversity hotspot and optimal habitat zone strongly supports the stability–diversity hypothesis, where climatically stable regions accumulate and maintain higher levels of genetic diversity [70,71]. The concentration of optimal habitats in Central and Eastern Anatolia is critically consistent with the genetic findings, identifying these regions as primary Pleistocene refugia with maximum genetic diversity. This correspondence between current habitat suitability and genetic diversity patterns supports the stability–diversity hypothesis and validates our integrated approach.

This finding emphasizes the species’ dependence on specific thermal regimes characteristic of continental steppe environments, where moderate temperatures during dry periods (15–25 °C) and consistent daily temperature fluctuations create optimal foraging and physiological conditions. The secondary importance of precipitation variables (bio14, bio19) reflects the species’ adaptation to arid environments, where the moisture availability during critical periods constrains population persistence.

The sharp habitat suitability peak at intermediate temperatures during dry periods, combined with the secondary peak under colder conditions (−6 to 1 °C), may reflect a population structure corresponding to the Eastern Anatolian highland populations versus Central Anatolian steppe populations. This bimodal niche pattern might supports the genetic evidence for multiple refugial areas with distinct environmental adaptations, consistent with Middle Pleistocene divergence patterns where subspecific lineages separated 600–800 thousand years ago [14,70,72]. The combination of these climatic variables defines optimal habitat conditions that have remained relatively stable, explaining both the existing concentration of genetic diversity and the historical refugial role of this region.

### 4.4. Biogeographic Barriers and Isolation by Distance

The strong isolation by distance pattern (r = 0.735, *p* < 0.001) operating across the species’ approximate 2500 km range provides crucial insights into the dispersal limitation in small mammal populations, as IBD patterns directly reflect the balance between the genetic drift and gene flow mediated by a limited dispersal capacity [73,74,75]. The hierarchical nature of this pattern, with stronger isolation between Iran–Türkiye (reflecting deep evolutionary divergence predating the Early Pleistocene) than among Turkish regional populations (reflecting more recent Middle Pleistocene differentiation), suggests that biogeographic barriers of different ages and strengths have shaped the population connectivity across the range [76]. This geographic scale over which the IBD is detected suggests that the species has a limited long-distance dispersal capacity, which may influence its ability to track suitable habitats under future climate change scenarios.

The Anatolian Diagonal emerges as a particularly significant climatic transition zone, consistently appearing as an important feature in the habitat distribution under all climate scenarios. This northeast–southwest trending transition has been documented as a major influence on species distributions across multiple taxa, creating a gradient between humid northern and western regions and the continental interior steppes [1,6]. Populations on either side of the Anatolian Diagonal show distinct genetic clustering, indicating that this climatic transition has shaped the genetic structure of *S. williamsi*. However, the mixed phylogenetic patterns in Central Anatolian populations suggest this region may have served as a dispersal corridor connecting eastern and western refugia during the late Early Pleistocene radiation of the *S. euphraticus* species group [14].

### 4.5. Climate Change Vulnerability and Range Dynamics

The species distribution modeling reveals concerning patterns of habitat contraction under future climate scenarios, with projected losses ranging from 63.69% under the optimistic SSP1-2.6 scenario to 98.41% under SSP3-7.0 by 2081–2100. These projections are particularly alarming given the species’ demonstrated dispersal limitations and strong isolation by distance patterns, which suggest a limited capacity for rapid range tracking [77,78].

The sharp declines in suitability beyond optimal ranges revealed by the niche modeling suggest that *S. williamsi* is highly vulnerable to climate change, contradicting the intuitive expectation that open landscape specialists might benefit from global warming. Desert-adapted small mammals often inhabit environments near their physiological tolerance limits for temperature and aridity, making them paradoxically vulnerable to further warming [19,79]. The niche model demonstrated the species’ sensitivity to climatic extremes, under changing environmental conditions.

Under the most optimistic scenario (SSP1-2.6), suitable habitats are projected to remain relatively stable within Central Anatolia, with an eastward expansion and persistence in interior steppe regions. However, under higher emission scenarios (SSP2-4.5, SSP3-7.0, SSP5-8.5), results suggest severe range contractions with projected decreases in suitability across most regions. The eastward habitat shifts projected under all scenarios may be constrained by the Anatolian Diagonal, limiting the species’ ability to track suitable climates through altitudinal or latitudinal migration.

### 4.6. Comparative Phylogeography and Regional Patterns

The phylogeographic pattern observed in *S. williamsi* contributes to our understanding of Anatolian biogeography while revealing some unique characteristics. The east-to-west diversity gradient, with the highest nucleotide diversity in Eastern Anatolia declining westward to the Aegean region, parallels patterns documented in other regional endemics and supports models of eastward refugial persistence during glacial maxima [80,81]. The phylogenetic reconstruction suggests that the primary dispersal of *S. williamsi* occurred from east to west, consistent with the general pattern observed in many Anatolian taxa [32,62,81].

However, the extreme level of the population structure in *S. williamsi*, with no shared haplotypes between regions, is remarkable even by Anatolian standards. This may reflect the species’ semi-fossorial lifestyle and specific habitat requirements, which could result in particularly strong isolation compared to more vagile taxa [82]. The star-like phylogenetic patterns observed in some Western Anatolian clades are consistent with rapid population expansion, representing post-glacial colonization events that occurred well after the Early Pleistocene radiation that established the major lineage boundaries within the *S. euphraticus* species group [14]. This expansion pattern has been documented in numerous Anatolian endemic species and reflects the complex interplay between climatic fluctuations and topographic barriers [83].

### 4.7. Implications for Steppe Ecosystem Understanding

This study provides novel insights into steppe ecosystem dynamics by demonstrating how phylogeographic structures and species distribution modeling (SDM) can jointly inform climate vulnerability assessments of steppe-adapted fauna. The exceptionally high genetic diversity of *S. williamsi*, concentrated in Central and Eastern Anatolia, is consistent with the stability–diversity hypothesis, which predicts that long-term climatic stability promotes the accumulation and maintenance of genetic diversity [70,71]. Similar findings have been reported in the Brazilian Atlantic Forest [70] in extra-Mediterranean refugia across Europe [81], emphasizing that Central Anatolian steppes function as evolutionary refugia that preserve genetic variation in continental grassland species.

The projected severe habitat contractions for *S. williamsi* also align with broader SDM-based assessments of arid and steppe fauna. For instance, *Triturus newts* in Anatolia exhibited comparable refugial persistence combined with future vulnerability when SDM was integrated with mitochondrial phylogeography [32]. Likewise, desert plants, such as *Welwitschia mirabilis* in the Namib Desert (Bombi et al. 2021 [25]), and small mammal communities in North America (Moritz et al. 2008 [20]) have demonstrated heightened sensitivity to warming, illustrating that species already occupying the edge of their climatic tolerance are disproportionately threatened. The congruence between genetic structuring and habitat projections in *S. williamsi* underscores the utility of integrating SDM with phylogeographic data to identify both contemporary refugia and future extinction risks [30,84].

Combined with threats from agricultural intensification [85], the severe climate-driven range contractions predicted here suggest that Anatolian steppes represent both a global biodiversity hotspot and a climate-vulnerable biome, where multiple endemic species may face parallel declines [86,87]. The integration of genetic diversity patterns, SDM projections, and climate impact assessment in *S. williamsi* highlights Central Anatolia as a critical climate refugium. Protecting such refugial landscapes will not only safeguard evolutionary heritage but also buffer broader steppe ecosystem functions under accelerating global change.

## 5. Conclusions

This study aimed to investigate the phylogeographic structure and climatic vulnerability of *Scarturus williamsi* across its distribution range in Anatolia and adjacent regions by integrating genetic analyses with ecological niche modeling. We identified a hierarchical pattern of population divergence, with the deepest split between Iranian and Turkish lineages (>780,000 years ago) and a more recent divergence among Turkish regional populations (~440,000 years ago), based on temporal frameworks from Lebedev & Shenbrot [11]. This corresponds to the ancient isolation (>780,000 years ago) for Iran–Turkey versus more recent splits (~440,000 years ago) between Turkish regions.

The analysis reveals no shared haplotypes between any regions, representing extreme isolation even by Anatolian standards. The phylogeographic structure shows a perfect correspondence with species distribution models, demonstrating a remarkable climate–genetics alignment. The species exhibits bimodal climate preferences reflecting distinct highland versus steppe populations yet paradoxically shows severe vulnerability to climate change. The habitat loss projections represent the most alarming finding, revealing an emissions-dependent vulnerability gradient where even optimistic climate scenarios result in substantial range contraction (60–70% loss), while realistic scenarios project severe habitat reductions (>90% loss). The accelerating pattern of habitat degradation, with the steepest losses occurring in the mid-century across all scenarios, emphasizes the urgent need for immediate conservation action. Central Anatolian core areas demonstrate relative persistence, making them priority conservation targets for maintaining remnant populations under climate change.

The Mean Temperature of Driest Quarter (bio9) emerges as the primary driver of jerboa distributions across continental scales, explaining both current genetic patterns and future vulnerability. This demonstrates how ancient and recent evolutionary processes combine to create complex population structures, with important implications for predicting climate change responses in continental steppe fauna.

Nevertheless, our study is limited by the geographic scope of the sampling, potential biases in ecological niche models, and uncertainties in future climate scenarios. Future research should expand genomic sampling across unsampled areas, incorporate landscape connectivity models, and apply integrative approaches across multiple steppe species to refine predictions and conservation strategies.

## Figures and Tables

**Table 1:** Population genetic diversity statistics for *S. williamsi* across five populations (n = 98). N = sample size; Nh = number of haplotypes; Hd = haplotype diversity; π = nucleotide diversity; S = segregating sites; θw = Watterson’s theta; and Tajima’s D, with associated *p*-values testing deviation from neutrality.

Population	N	Nh	Hd	π	S	θw	Tajima’s D	*p*-Value
Eastern Anatolia	16	15	0.9917	0.023761	74	22.301	−0.231	0.817
Central Anatolia	55	50	0.9966	0.014861	104	22.730	−1.475	0.140
Iran–Azerbaijan	10	9	0.9778	0.016692	51	18.028	−0.869	0.385
Aegean Region	11	10	0.9818	0.007535	29	9.901	−1.500	0.134
Black Sea Region	6	6	1.0000	0.006907	16	7.007	−0.771	0.441

**Table 2:** Permutation-based relative variable importance for the *Scarturus williamsi* Random Forest SDM, evaluated using Pearson correlation and AUC metrics.

Variable	Description	Correlation (%)	AUC (%)
bio2	Mean Diurnal Range (°C)	16.2	5.2
bio3	Isothermality (%)	9.6	2.4
bio4	Temperature Seasonality (standard deviation × 100)	4.5	1.4
bio8	Mean Temperature of Wettest Quarter (°C)	6.6	1.9
bio9	Mean Temperature of Driest Quarter (°C)	23.5	8.6
bio14	Precipitation of Driest Month (mm)	10.0	5.2
bio15	Precipitation Seasonality (coefficient of variation)	9.9	3.9
bio19	Precipitation of Coldest Quarter (mm)	3.4	1.1

**Table 3:** Projected habitat loss for *S. williamsi* across four time periods under climate change scenarios. Values represent percentage of habitat loss relative to current suitable habitat extent, calculated using ensemble species distribution models with a habitat suitability threshold of 0.3 (≥30% suitability).

Time Period	SSP1-2.6	SSP2-4.5	SSP3-7.0	SSP5-8.5
2021–2040	48.24	46.92	39.76	43.46
2041–2060	65.39	69.17	73.82	81.77
2061–2080	67.81	86.55	79.71	97.34
2081–2100	63.69	90.27	98.41	93.14

## Data Availability

All data used in this study were obtained from publicly available sources. Genetic sequence data are listed in the Supplementary Materials and were retrieved from the GenBank database. No new datasets were generated during the current study.

## Supplementary Materials

The following supporting information can be downloaded at https://www.mdpi.com/article/10.3390/biology14091184/s1: Figure S1: Alignment of 98 *Scarturus williamsi* cytochrome b sequences, with nucleotide positions scaled to 888 bp; Figure S2: Relative importance of bioclimatic variables in the species distribution model for *Scarturus williamsi*; and Table S1: GenBank accession numbers and geographic origins of 98 *Scarturus williamsi* cytochrome b sequences used in the study.
