# Supplementary material for: Phylogeography of Scarturus williamsi and Climate Change Impacts: Genetic Diversity and Projected Habitat Loss in Anatolia
Source: Biology (Basel). 2025 Sep 3;14(9):1184. doi: 10.3390/biology14091184 (PMC12467805; doi:10.3390/biology14091184)
Supplement: Supplementary file 1 [file biology-14-01184-s001.zip › biology-3797778-supplementary.pdf]

Supplementary Figures

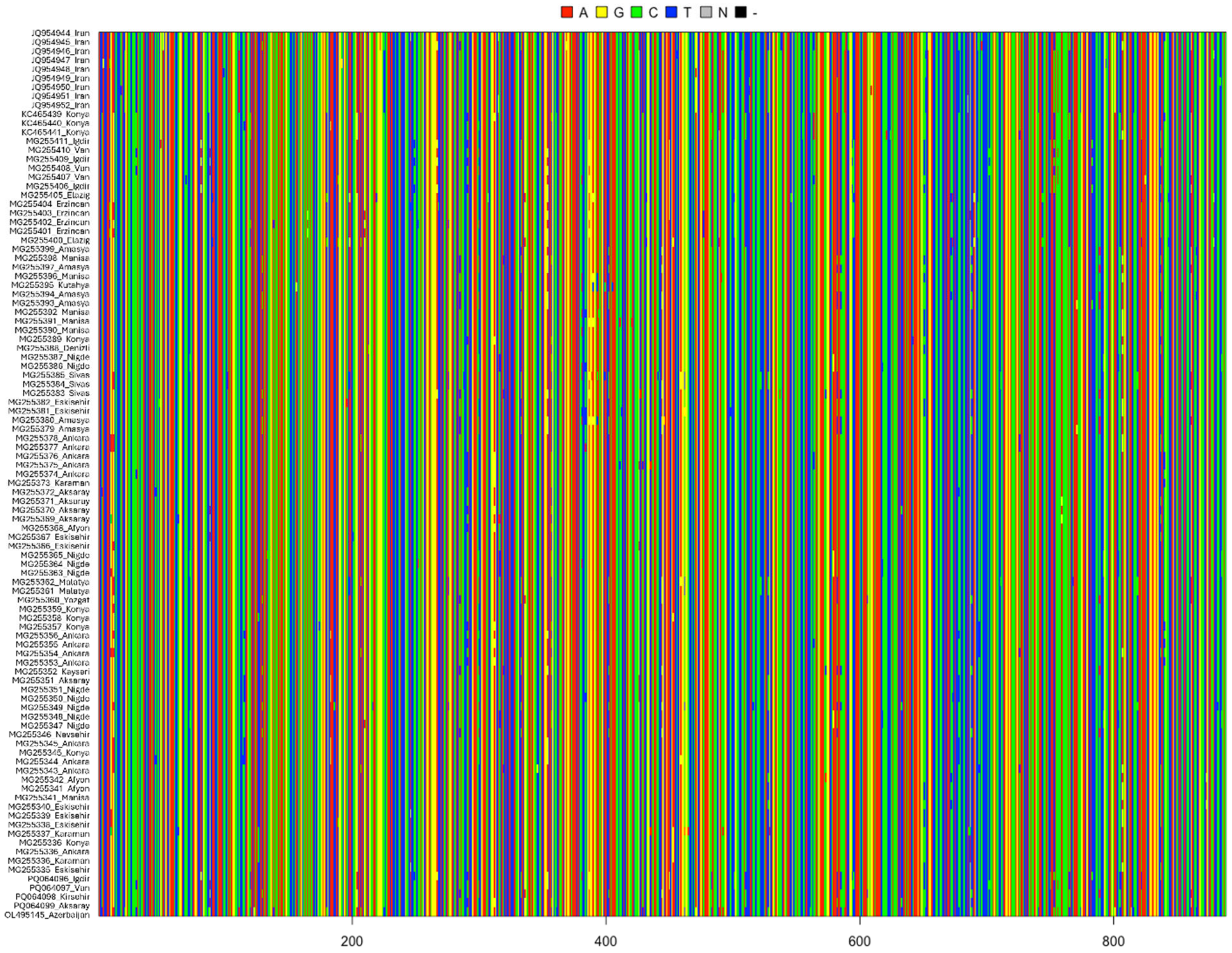

Figure S1: Alignment of 98 *Scarturus williamsi* cytochrome b sequences, with nucleotide positions scaled to 888 bp

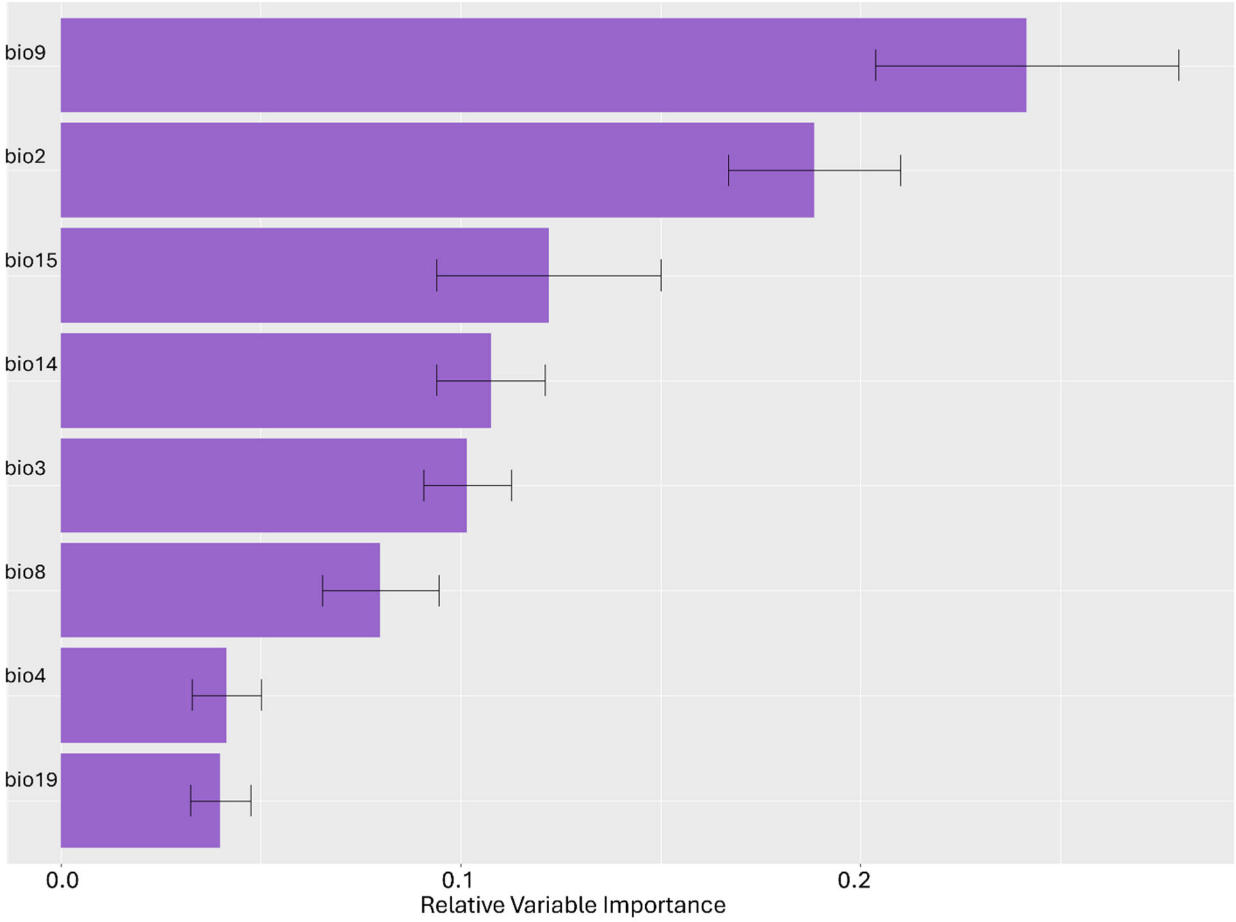

Figure S2: Relative importance of bioclimatic variables in the species distribution model for *Scarturus williamsi.* Importance values represent the proportional contribution of each variable to model predictive accuracy, determined through permutation-based analysis. Error bars show standard deviation across model replicates. Variables are ranked by importance from highest (bio9) to lowest (bio19). See Table 3 for detailed variable descriptions and quantitative importance values.

Supplementary Table

Table S1: GenBank accession numbers and geographic origins of 98 *Scarturus williamsi* cytochrome b sequences used in the study

Accsesion No	Location	Region
**MG255388**	Denizli	Aegean Region
**MG255368**	Afyon	Aegean Region
**MG255342**	Afyon	Aegean Region

MG255341	Afyon	Aegean Region
MG255395	Kutahya	Aegean Region
MG255398	Manisa	Aegean Region
MG255396	Manisa	Aegean Region
MG255392	Manisa	Aegean Region
MG255391	Manisa	Aegean Region
MG255390	Manisa	Aegean Region
MG255341	Manisa	Aegean Region
MG255399	Amasya	Black Sea Region
MG255397	Amasya	Black Sea Region
MG255394	Amasya	Black Sea Region
MG255393	Amasya	Black Sea Region
MG255380	Amasya	Black Sea Region
MG255379	Amasya	Black Sea Region
MG255372	Aksaray	Central Anatolia
MG255371	Aksaray	Central Anatolia
MG255370	Aksaray	Central Anatolia
MG255369	Aksaray	Central Anatolia
MG255351	Aksaray	Central Anatolia
PQ064099	Aksaray	Central Anatolia
MG255378	Ankara	Central Anatolia
MG255377	Ankara	Central Anatolia
MG255376	Ankara	Central Anatolia
MG255375	Ankara	Central Anatolia
MG255374	Ankara	Central Anatolia
MG255356	Ankara	Central Anatolia
MG255355	Ankara	Central Anatolia
MG255354	Ankara	Central Anatolia
MG255353	Ankara	Central Anatolia
MG255345	Ankara	Central Anatolia
MG255344	Ankara	Central Anatolia
MG255343	Ankara	Central Anatolia
MG255336	Ankara	Central Anatolia
MG255382	Eskisehir	Central Anatolia
MG255381	Eskisehir	Central Anatolia
MG255367	Eskisehir	Central Anatolia
MG255366	Eskisehir	Central Anatolia
MG255340	Eskisehir	Central Anatolia
MG255339	Eskisehir	Central Anatolia
MG255338	Eskisehir	Central Anatolia
MG255335	Eskisehir	Central Anatolia
MG255373	Karaman	Central Anatolia
MG255337	Karaman	Central Anatolia
MG255336	Karaman	Central Anatolia
MG255352	Kayseri	Central Anatolia
PQ064098	Kirsehir	Central Anatolia

KC465439	Konya	Central Anatolia
KC465440	Konya	Central Anatolia
KC465441	Konya	Central Anatolia
MG255389	Konya	Central Anatolia
MG255359	Konya	Central Anatolia
MG255358	Konya	Central Anatolia
MG255357	Konya	Central Anatolia
MG255345	Konya	Central Anatolia
MG255346	Nevsehir	Central Anatolia
MG255387	Nigde	Central Anatolia
MG255386	Nigde	Central Anatolia
MG255365	Nigde	Central Anatolia
MG255364	Nigde	Central Anatolia
MG255363	Nigde	Central Anatolia
MG255351	Nigde	Central Anatolia
MG255350	Nigde	Central Anatolia
MG255349	Nigde	Central Anatolia
MG255348	Nigde	Central Anatolia
MG255347	Nigde	Central Anatolia
MG255385	Sivas	Central Anatolia
MG255384	Sivas	Central Anatolia
MG255383	Sivas	Central Anatolia
MG255360	Yozgat	Central Anatolia
MG255405	Elazig	Eastern Anatolia
MG255400	Elazig	Eastern Anatolia
MG255404	Erzincan	Eastern Anatolia
MG255403	Erzincan	Eastern Anatolia
MG255402	Erzincan	Eastern Anatolia
MG255401	Erzincan	Eastern Anatolia
MG255411	Igdir	Eastern Anatolia
MG255409	Igdir	Eastern Anatolia
MG255406	Igdir	Eastern Anatolia
PQ064096	Igdir	Eastern Anatolia
MG255362	Malatya	Eastern Anatolia
MG255361	Malatya	Eastern Anatolia
MG255410	Van	Eastern Anatolia
MG255408	Van	Eastern Anatolia
MG255407	Van	Eastern Anatolia
PQ064097	Van	Eastern Anatolia
OL495145	Azerbaijan	Iran-Azerbaijan
JQ954944	Iran	Iran-Azerbaijan
JQ954945	Iran	Iran-Azerbaijan
JQ954946	Iran	Iran-Azerbaijan
JQ954947	Iran	Iran-Azerbaijan
JQ954948	Iran	Iran-Azerbaijan
JQ954949	Iran	Iran-Azerbaijan

**JQ954950**	Iran	Iran-Azerbaijan
**JQ954951**	Iran	Iran-Azerbaijan
**JQ954952**	Iran	Iran-Azerbaijan
